# An ultrasonic degraded polysaccharide extracted from *Pueraria lobata* ameliorate ischemic brain injury in mice by regulating the gut microbiota and LPS-TLR4 pathway

**DOI:** 10.1016/j.ultsonch.2024.107200

**Published:** 2024-12-13

**Authors:** Yulong Zhang, Zuman Dou, Shanshan Li, Huaying Zhang, Shanshui Zeng, Xiangyu Zuo, Yu Xiao, Lingling Zhang, Zhixin Li, Qingfeng Zhu, Wenyang Zhang, Hui Niu, Qingfei Duan, Xiaoxia Chen, Zhuang Li, Hongwei Zhou, Qian Wang

**Affiliations:** a Microbiome Medicine Center, Department of Laboratory Medicine, Zhujiang Hospital, Southern Medical University, Guangzhou 510282, China; b College of Light Chemical Industry and Materials Engineering, Shunde Polytechnic, Foshan 528333, China; c School of Food Science and Engineering, South China University of Technology, Guangzhou 510640, China; d Nutritional and Food Science Research Institute, Department of Nutrition and Food Hygiene, School of Public Health, Shanxi Medical University, Taiyuan 030001, China

**Keywords:** Ultrasonic degradation, *Pueraria lobata* polysaccharide, Ischemic brain injury, Gut microbiota, LPS/TLR4/MyD88 pathway

## Abstract

Ischemia brain injury is closely associated with the gut microbiota. Polysaccharides, as a typical prebiotic, have been extensively employed in stroke treatment. In our previous study, *Pueraria lobata* polysaccharide (PLP-3) with antioxidant activity was prepared via water extraction and alcohol precipitation combined with ultrasonic degradation. In this study, the effects of PLP-3 on ischemia brain injury and its regulatory effects on the gut microbiota were further investigated. The results demonstrated that PLP-3 effectively reduced the infarct area, improves neurological function, and alleviates neuronal damage of cerebral ischemia injury. Mechanistically, PLP-3 significantly reduces serum LPS levels in MCAO mice, inhibiting TLR-4 activation in brain tissue and thereby reducing IL-1β and TNF-α levels. Meanwhile, PLP-3 significantly repaired the intestinal barrier injury by increasing the expression of tight junction proteins (ZO-1 and Occludin) and increasing the number of goblet cells. Additionally, the structure and composition of gut microbiota in MCAO mice after PLP-3 intervention, were also significantly changed, especially the enrichment of *Lactobacillus* and the reduction of *Corynebacterium* and *Staphylococcus*. At the same time, short chain fatty acid, metabolites of gut microbiota, were also significantly increased and significantly correlated with the abundance of *Lactobacillus*. Moreover, LC-MS untargeted metabolomics revealed that PLP-3 significantly improves the intestinal metabolic profile after cerebral ischemia injury, upregulating the amino acid biosynthesis pathway and enriching amino acids such as glutamine and arginine, as well as neuroprotective flavonoids such as fisetin and liquiritigenin. These results suggested that PLP-3 could protect mice from cerebral ischemia–reperfusion injury by regulating gut microbiota and repairing gut barrier, inhibiting brain LPS/TLR4/MyD88 inflammatory pathway, therefore we provide a theoretical basis for PLP-3 as a functional food to prevent ischemic brain injury.

## Graphical abstract

**Figure:**
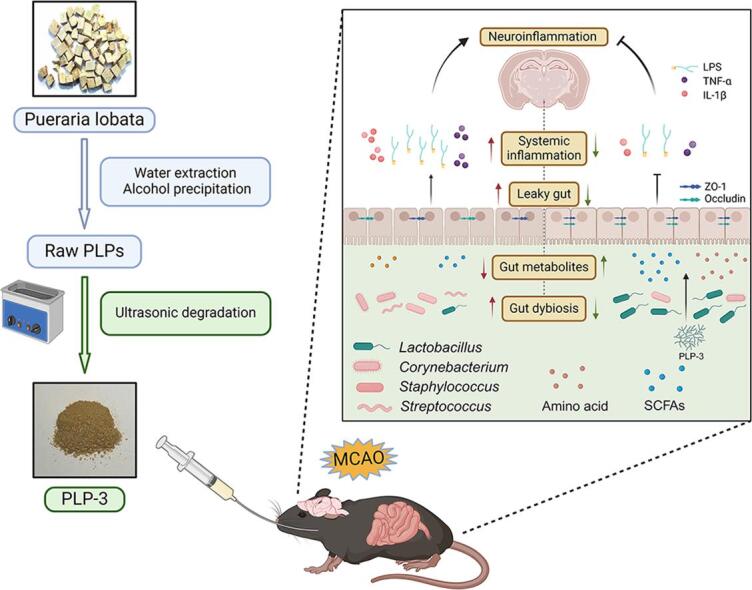

## 1 Intoduction

Stroke is the second leading cause of death and disability globally[1], It is primarily classified into ischemic and hemorrhagic stroke, with ischemic stroke accounting for 85 % of cases[2], [3]. Ischemic stroke occurs when insufficient blood flow to the brain results from stenosis or occlusion of the cerebral blood supply arteries (carotid artery and vertebral artery), leading to brain tissue necrosis and triggering a cascade of pathological processes in the affected brain region[4], including oxidative stress, neuroinflammation, and apoptosis[5]. Thrombolysis and mechanical thrombectomy are the primary therapeutic modalities for ischemic stroke. However, these interventions are associated with an increased risk of bleeding and other complications. Furthermore, they may not prevent recurrent strokes or fully restore neurological function, while associating with substantial costs[6]. Therefore, novel therapeutic modalities are urgently needed to address the unmet clinical need in the treatment of cerebral ischemia.

The intestinal barrier is primarily composed of three components: the mucosal barrier, mechanical barrier, and microbial barrier[7]. Emerging evidence suggests a strong association between intestinal barrier damage and gut microbiota dysbiosis with the occurrence and progression of stroke. Experiments has demonstrated that after ischemic stroke, sympathetic nerve excitation can induce intestinal ischemia and hypoxia injury, leading to damage to the intestinal tight junction, translocation of gut microbiota and disrupted intestinal immune homeostasis, with the migration of intestinal-derived T cell[8], [9], [10]. Notably, cohort analysis of ischemic stroke patients has revealed rapid changes in gut microbiota composition, with an enrichment of opportunistic pathogens such as *Enterobacteriaceae*, *Megasphaera*, and *Oscillibacter*, and a depletion of probiotics such as *Lactobacillus*, *Butyricicoccus*, *Meganonas*, *Prevotella*, and *Alloprevotella*[11], [12]*.* Probiotic, such as *Lactobacillus rhamnosus*, has been reported to exert neuroprotective effects by reducing neuroinflammation in the brain after ischemia[13]. However, due to the bactericidal effects of digestive juices, the colonization rate of directly administered probiotics is variable.

Furthermore, gut microbiota dysbiosis following stroke is also associated with metabolic disturbances, including alterations in bile acids, short-chain fatty acids (SCFAs) and animo acid metabolism[14]. SCFAs disorder is observed both in human and mice with cerebral ischemia injury, SCFAs like butyrate have been shown to ameliorate poststroke cognitive impairment in mice[15], transplantation the fecal with high level of SCFAs also protect SD rats from cerebral ischemia, and improve the intestinal barrier[16]. Amino acid metabolism is closely related to the intestinal barrier and nervous system homeostasis, tryptophan and its microbial metabolites, such as indole-propionic acid, regulate immune homeostasis in distant target organs via the aryl hydrocarbon receptor[17], L-arginine is used clinically as a nitric oxide donor to treat mitochondrial encephalopathy and ischemic stroke[18]. Therefore, targeting the improvement of gut dysbiosis after stroke has great potential as a therapeutic strategy for future stroke treatment.

Polysaccharides are long-chain polymers composed of multiple monosaccharides connected by glycosidic bonds. They are important components of dietary fiber in plants and are difficult to degrade and utilize by endogenous enzymes in the human body[19], [20]. However, gut microbiota can actively respond to dietary polysaccharides due to their rich repertoire of carbohydrate-degrading enzymes, degrading polysaccharides and producing beneficial substances such as SCFAs[19], [21]. Previous studies have shown that dietary polysaccharides from different sources have different regulatory effects on the gut microbiota. *Pericarpium Citri Reticulatae ‘Chachiensis’* polysaccharides increased the abundance of *Parabacteroides goldsteinii* [22], while Polysaccharides *from Lyophyllum decastes* enriched the probiotic strains *Lactobacillus johnsonii* and *Bacteroides intestinalis*[23]. Supplementation with a synbiotic containing *Lactobacillus reuteri* and fructooligosaccharides can improve brain ischemic injury through regulating the gut-brain axis by promoting specific proliferation of *Lactobacillus reuteri* and *Prevotella copri*[24].

*Pueraria lobata*, a plant with both medicinal and edible properties, is widely used in traditional Chinese medicine to treat cardiovascular and cerebrovascular diseases, hypertension, hyperlipidemia, coronary heart disease, and other ailments[25], [26]. Polysaccharides are the main active components in *Pueraria lobata* and exhibit diverse biological activities, including antioxidant, hypoglycemic, and gut microbiota regulation[27]. In a prior study, we employed a combination of water extraction, alcohol precipitation, and ultrasonic degradation to prepare *Pueraria lobata* polysaccharides with varying molecular weights (PLP, PLP-1, PLP-3, and PLP-7). Subsequent comparison of their structural characteristics and antioxidant activities led to the selection of PLP-3 (obtained after 3 h of ultrasonic degradation) for further investigation. PLP-3 is primarily composed of glucose (98 %), along with galacturonic acid, galactose, and xylose, and possesses a molecular weight of 103.08 kDa. Compared to polysaccharides prepared solely by water extraction and alcohol precipitation, PLP-3 exhibits enhanced efficacy in alleviating palmitic acid-induced hepatic lipid metabolism disorders and oxidative stress damage, demonstrating superior hepatoprotective effects[28]. Moreover, Lian et al. found that *Pueraria* resistant starch can enrich beneficial bacteria at the genus level in the gut, such as *Akkermansia* and *Bifidobacterium*, it also exerts neuroprotective effects after stroke by increasing melatonin levels in the brain[29]. Nevertheless, the neuroprotective potential of bioactive polysaccharides from *Pueraria lobata* against stroke-induced brain injury and their modulatory effects on the gut microbiota warrant further exploration.

Therefore, the present study aims to investigate the protective effects of PLP-3 on brain injury through the brain-gut axis. Using behavioral experiments and molecular biology experiments, we assess neurological function and intestinal barrier integrity recovery in MCAO mice after intervention with PLP-3. Additionally, we explore the effects of PLP-3 on the gut microbiota and metabolites after ischemic brain injury by a combination of 16S rRNA sequencing, targeted SCFAs profile and non-targeted metabolomics. The results of this study will provide a scientific basis for developing intestinal microbiota-based health food products to improve stroke prognosis.

## 2 Materials and methods

### 2.1 Materials and reagents

The dried root of *Pueraria lobata* was purchased from Guangzhou Zhixin Pharmaceutical Co., LTD (Guangzhou, China). 2,3,5-triphenyltetrazolium chloride (TTC) staining were obtained from Sigma-Aldrich Chemical Co. (MO, USA). Monofilament nylon sutures were purchased from Jialing Biotechnology (Guangzhou, China). Enzyme-linked immunosorbent assay (ELISA) kits for lipopolysaccharide (LPS), Inflammatory cytokines interleukin-1β (IL-1β) and tumor necrosis factor α (TNF-α) were purchased from the Meimian (Nanjing, China). Western blotting antibodies: TLR-4, Myd88, ZO-1, Occludin, β-Actin and horseradish peroxidase conjugated secondary antibodies were all purchased from Cell Signaling Technology (MA, USA). All other chemicals and reagents were of analytical grade.

### 2.2 Extraction of Pueraria lobata polysaccharides (PLP)

The PLP was prepared and purified according to our published method. Briefly, the dried root of Pueraria lobata was ground into powder and defatted with 95 % ethanol at 70℃ for three times. The dried defatted powder was then extracted with deionized water (1:20, m/v) at 90℃ for two times. The supernatant was collected and concentrated under reduced pressure, then precipitated with ethanol (1:4, v/v) at 4℃ overnight. The precipitate was redissolved in deionized water, then deproteinized with 20 % trichloroacetic acid (1:1, v/v) and decolored with AB-8 macroporous resin. Finally, the solution was dialyzed (cut-off: 3500 Da) against deionized water at 4℃ for 72 h, and lyophilized to obtain PLP.

### 2.3 PLP-3 was prepared by ultrasonic degradation

Ultrasonic degradation was conducted using an ultrasonic homogenizer (JY92-ⅡN, Ningbo Scientz Biotechnology, Ningbo, China) at a frequency of 20 kHz according to reported method. 100 mL of PLP solution (10 mg/mL) was treated with ultrasound in pulse mode (2 s on, 2 s off) for 3 h. The temperature of solution was kept at 25 ± 1℃ and ultrasonic power was set as 650 W. After ultrasonic treatment, the PLP solution was collected, lyophilized and named as PLP-3.

### 2.4 Animals and animal experiment

The mice used in this experiment were 8–10 weeks old wild-type C57 BL/6J male mice, purchased from Zhuhai BesTest Bio-Tech (Zhuhai, China), and bred in the SPF-level experimental animal house of Zhujiang Hospital, Southern Medical University (Guangzhou, China). The breeding environment temperature was maintained at 25 ± 1°C, with regular lighting (12 h/12 h light/dark), high-pressure water and regular SPF-grade mouse feed. All animal experiments were approved by the Ethics Review Committee of the Experimental Animal Center of Zhujiang Hospital, Southern Medical University (LAEC-2024–016). Animal welfare and experimental programs strictly comply with the guidelines for the care and use of laboratory animals.

**Fig. 1:**
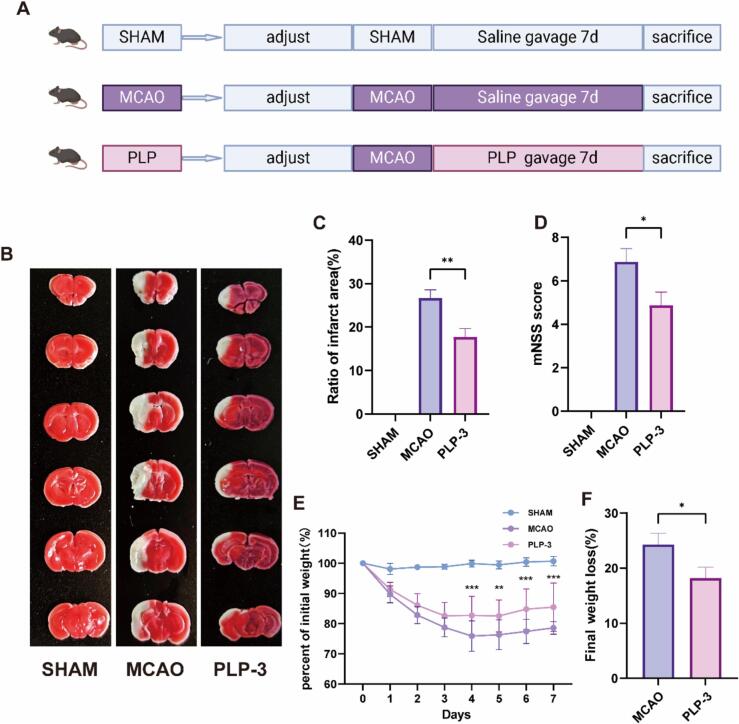
PLP-3 improves MCAO mice brain injury and overall outcome (n = 8). (A) The schematic of animal experiment, (B-C) Brain infarction area, (D) mNSS score, (E-F) body weight loss.

For PLP-3 treatment experiment, 48 male C57 BL/6J mice were randomly divided into three groups (n = 16/group): sham-operated (SHAM), middle cerebral artery occlusion (MCAO), and PLP-3 treatment. Mice in the SHAM group underwent vascular isolation and suture and were intragastrically administered with saline daily for 7 days. Mice in the MCAO group underwent MCAO surgery and were intragastrically administered with saline daily for 7 days. Mice in the PLP-3 group underwent MCAO surgery and were intragastrically administered with 400 mg/kg PLP-3 daily for 7 days. Blood, brain, intestine tissue, and intestinal contents were collected from all groups during dissection, the experimental design is shown in Fig. 1A.

### 2.5 Establishment of the MCAO mice model

The cerebral ischemic model was prepared according to a previously described method[30]. Mice were anesthetized with 1.25 % tribromoethanol (0.2 ml/10 g) via intraperitoneal injection. Under microscopic visualization, the right internal, external, and common carotid arteries were carefully dissected and ligated. A monofilament nylon suture was inserted into the bifurcation of the internal carotid artery from the external carotid artery. After 1 h of ischemia, the nylon suture was removed, and the suture knot around the common carotid artery was loosened to induce reperfusion. Mice with a score of 2 points on the Zea Longa method scoring system were considered to be successfully modeled and were included in the subsequent experiment[31].

### 2.6 mNSS score and TTC staining

Neurological outcomes were blindly assessed by an investigator using the modified Neurological Severity Score (mNSS) at 7 days post-MCAO[32]. The mNSS is a composite of motor, sensory, reflex, and balance tests, with scores ranging from 0 to 14, where higher scores indicate more severe brain injury.

Cerebral infarction volume was measured in each group using TTC staining. Briefly, brain tissues were cut into 1 mm-thick coronal sections. The sections were then stained with TTC at 37 °C for 20 min. Next, the sections were fixed in 4 % paraformaldehyde for 24 h, and images of the stained sections were acquired for analysis. ImageJ software was used to calculate cerebral infarction volume. Infarction volume ratio = (contralateral hemisphere − ischemic side non infarcted area)/ (contralateral hemisphere × 2) × 100 %, this formula corrects for the bias caused by cerebral edema on the infarcted side.

### 2.7 Open field test

Open field test is conducted following the method as described[33]. The experimental setup consisted of a box (50 cm × 50 cm × 50 cm) and an automatic data acquisition processing system (Noduls Information Technology, Wageningen, Holland). Each mouse was placed in the center of the box, and the timer was started. The mouse was observed for 10 min, and the video tracking system recorded the video. Software was used to quantitatively analyze spontaneous movement, recording the total distance traveled by the mouse, entries into the center square and the percentage of time spent in the center square (stay time = time spent in the center square (s) / total time (s) × 100 %). Each mouse was tested individually and only once. The box was thoroughly cleaned and disinfected before each animal was tested.

### 2.8 HE and Nissl staining

Mouse whole brain and colon tissues were harvested and fixed in 4 % formalin, embedded in paraffin, and were cut into 5-µm-thick sections for staining with hematoxylin–eosin (H&E) or Alcian blue solutions (Solarbio, Beijing, China). For Nissl staining, brain sections were immersed in Nissl solution (Solarbio, Beijing, China) at 37 °C in an incubator for 30 min and dehydrated through a graded series of ethanol (70 %, 80 %, and 90 % ethanol, and twice in 100 % ethanol, each for 30 s). Apoptotic cells were counted using ImageJ software. Histological scores were evaluated according to the method reported by Lian et al.[29].

### 2.9 Measurement of serum inflammatory factor

After the sacrifice of mouse, blood was obtained and centrifuged at 3,500 rpm for 10 min at 4 °C. Serum was obtained for the detection of the inflammatory cytokines. LPS, IL-1β and TNF-α were determined using ELISA kits in accordance with the manufactures’ instructions.

### 2.10 qPCR analysis

Total RNA from mice tissue was separated using TRIzol reagent, and cDNA was synthesized by reverse transcription with a Prime Script RT reagent Kit (Accurate biology, Changsha, China). q-PCR was conducted under the following conditions: 95℃ for 1 min, 40 cycles of 95 °C for 15 s, followed by 60 °C for 1 min, using the SYBR Green Kit (Vazyme, Nanjing, China) on the ABI Step One RT-PCR system (Applied Biosystems, Foster City, USA). The relative expression levels were calculated using the 2^−ΔΔCt^ method using β-Actin for normalization. The primers were designed as shown in Table 1.

**Table 1:** The primer sequence.

Primer name		Primer sequences (5′-3′)
IL-1β	F	TGCCACCTTTTGACAGTGATG
	R	TGATGTGCTGCTGCGAGATT
TNF-α	F	ATGGCCTCCCTCTCATCAGT
	R	TTTGCTACGACGTGGGCTAC
IL-6	F	CCCCAATTTCCAATGCTCTCC
	R	CGCACTAGGTTTGCCGAGTA
ZO-1	F	TTCACGCAGTTACGAGCAA
	R	TTGGTGTTTGAAGGCAGAGC
Occludin	F	TCTTTCCTTAGGCGACAGCG
	R	AGATAAGCGAACCTGCCGAG
β-Actin	F	GAGGGGCAATTTAGGGTGGG
	R	TCAAGACCGAAGGTGGGGTA

### 2.11 Western blot

Western blot experiments were carried out following our previous research[34]. Brain tissues from ischemic penumbra and colon tissue were lysed with cytoplasmic extraction reagents (Beyotime Biotechnology, China) containing protease inhibitor (Beyotime Biotechnology, China) using homogenizer and then centrifuged at 14,000 g for 30 min at 4 ◦C. Supernatant was collected and protein concentrations were assessed using the BCA assay (KeyGen Biotech, Nanjing, China). Equal amounts of protein (50μg) were separated by 10 % SDS–PAGE for electrophoresis and then transferred into nitrocellulose membranes (Millipore, CA, USA). The membranes were blocked with 5 % skim milk for 1 h at 37 ℃ and then incubated with the primary antibodies like anti-Toll-like receptor 4 (1:1000), anti-Myd88 (1:1000), anti-ZO-1 (1:1000), anti-Occludin (1:2000), anti-β-Actin (1:2000) at 4℃ overnight. Then, the membrane was then washed third in Tris buffered saline with Tween 20 (TBST) and incubated with a horseradish peroxidase conjugated secondary antibodies (1:2000) for 1 h at 37 ℃. The membrane was then visualized ECL Plus chemiluminescence reagent kit (Biosharp, Beijing, China) and quantified using ImageJ software.

### 2.12 16 s RNA sequence

The intestinal contents collected freshly and snap-frozen in liquid nitrogen before storage at − 80 °C in each treatment group was submitted to Personalbio Biotechnology (Shanghai, China) for high throughput sequencing within 16 S rRNA V3V4 hypervariable region. In general, the total bacterial genomic DNA of fecal samples was extracted by omega soil DNA kit (M5635-02) (Omega Bio-Tek, Norcross, GA, USA) according to the instructions. The collected DNA samples were identified by 1.20 % agarose gel electrophoresis and NanoDropNC2000 ultraviolet spectrophotometer for DNA concentration and quality. The sample volume was 5 μL, and the DNA concentration and OD260/280 ratio were detected. Forward primer 502F (5′-AYTGGGYDTAAAGNG-3′) and reverse primer 802R (5′-TACNVGGGTATCTAATCC-3′) were used to amplify by PCR within the V3V4 region of the above extracted DNA. The amplification system volume was 25 μL, including 5 μL 5 × reaction buffer, 5 μL5 × GC buffer, 2 μL dNTP (2.5 mM), 1 μL forward-primer (10 μM), 1 μL reverseprimer (10 μM), 2 μL DNA template, 8.75 μL ddH2O, and 0.25 μL Q5 DNA polymerase. The amplification products were cut to the same size as the Marker of 500 bp to obtain the target fragments by agarose gel electrolysis. The recovered products were quantified using the Quant-iT PicoGreen dsDNA assay kit (Invitgen, Carlsbad, CA, USA) and carried out on the Novaseq-PE250 platform for sequencing performance. Gut microbiota was analyzed 16S rDNA amplicon sequencing based on Personalbio platform in control and MCAO mice with or without PLP-3 supplementation.

### 2.13 Measuerment of SCFAs

In this study, SCFAs were quantified in fecal samples using a modified version of Lai's method[35]. Briefly, 100 mg of feces were suspended in 0.5 mL ultrapure water and ground using beads (1:5 feces:water, w/v) for 2 min (60 Hz). Subsequently, the samples were subjected to ultrasonic extraction in an ice bath for 10 min and vortexed for 2 min. Following incubation in the ice bath for 20 min, the supernatant was collected by centrifugation at 12,000 rpm for 20 min at 4 °C. The supernatant was then filtered through a 0.45 μm aqueous filter membrane for gas chromatography (GC) analysis.

GC analysis was performed using an Agilent 7820A GC system equipped with a flame ionization detector (FID) and an autosampler. The GC column (AE. FFAP, Lanzhouzhongkeantai Analytical Technology, China) measured 30 m in length, 0.25 mm in internal diameter, and had a film thickness of 0.50 μm. Nitrogen served as the carrier gas, flowing at a rate of 30 mL/min with a split ratio of 1:10. The oven temperature was initially maintained at 100 °C for 0.5 min, then increased to 160 °C at a rate of 4 °C/min. The temperature was then further increased to 220 °C at a rate of 10 °C/min and held for 2 min. The FID and injection port temperatures were set to 250 °C and 240 °C, respectively. Hydrogen and air flow rates were maintained at 40 mL/min and 400 mL/min, respectively. The injection volume was 1 μL, and the analysis time for each sample was 23.5 min.

### 2.14 Untargeted metabolomics

Cecal contents are thawed and mixed with a solvent to extract the metabolites. Sample separation was performed using an UHPLC (1290 Infinity LC, Agilent Technologies) HILIC and RPLC. The column temperature was 25℃ and flow rate was 300 μL/min. A number of 2 μL sample was loaded. The samples were placed in a autosampler at 4℃. For avoiding the influence caused by instrument detecting signal fluctuation, samples were analyzed randomly. QC samples were inserted into the analysis queue to evaluate the system stability and data reliability during the whole experimental process.

The mobile phase of chromatography was composed of buffer A (Water + 25 mM ammonium acetate + 25 mM ammonium hydroxide) and buffer B (Acetonitrile). The gradient was 95 % B for 1 min and was linearly reduced to 65 % in 13 min, and then was reduced to 40 % in 2 min and kept at 40 % for 2 min, and then increased to 95 % in 0.1 min, with a 5 min re-equilibration period employed.

Samples were detected in both ESI positive and negative modes. Analyses were performed using an UHPLC coupled to a quadrupole time-of-flight (AB SCIEX TripleTOF 6600). The ESI source conditions following HILIC separation were set as follows: Ion Source Gas1 (Gas1) as 60, Ion Source Gas2 (Gas2) as 60, curtain gas (CUR) as 30, source temperature: 600℃, Ion Spray Voltage Floating (ISVF) 5500 V in positive mode, and −5500 V in negative mode. In MS only acquisition, the instrument was set to acquire over the *m*/*z* range 60–1000 Da, product ion scan *m*/*z* range 25–1000 Da, TOF MS scan accumulation time 0.20 s/spectra, product ion scan accumulation time 0.05 s/spectra. The product ion scan is acquired using information dependent acquisition (IDA) with high sensitivity mode selected. The collision energy (CE) was fixed at 35 eV with ± 15 eV. Declustering potential (DP) was set as ± 60 V. IDA was set as follows: Exclude isotopes within 4 Da, Candidate ions to monitor per cycle:6.

Data analysis was performed as follows: (1) OPLS-DA was performed to analyze the difference between groups. (2) The different metabolites between groups and the VIP scores were analyzed. (3) KEGG pathway were analyzed according to the information of the metabolite comparison to the KEGG compound ID. (4) Random forest analysis were used to score the importance of differential metabolites for the classification

### 2.15 Data statistics

Statistical analyses were performed using Prism 8.0 (GraphPad Software Inc., USA). All the data were expressed as mean ± SEM. Data were presented as the mean ± standard error of the mean (SEM). One-way analysis of variance (ANOVA) was employed to assess the significance of differences among the mean values of the test levels, followed by the Duncan and LSD tests for post hoc analysis. The normality was checked by Shapiro-Wilk and Kolmogorov-Smirnov test, while the homogeneity of variances was checked by Brown-Forsythe and Bartlett test. P < 0.05 was considered as significantly different.

## 3 Results and discussions

### 3.1 PLP-3 improves MCAO mice brain injury and neurological function

Middle cerebral artery occlusion (MCAO) is a widely used method for modeling ischemic stroke. It involves inserting a nylon suture into the middle cerebral artery via the common carotid artery. Successful MCAO in mice typically results in symptoms such as hemiplegia, cerebral infarction, weight loss, and long-term neurological deficits[5]. In this study, to investigate the effect of PLP-3 on the severity of cerebral infarction in MCAO mice, we performed TTC staining on brain slices of MCAO mice. The MCAO mice without any intervention had a cerebral infarction area of 26.7 %, after one week of PLP-3 intervention, the cerebral infarction area of MCAO mice was significantly improved, and the infarction area was reduced to 17.7 % (Fig. 1B-C). The mNSS score was used to evaluate the neurological function of each group of mice. The neurological function of MCAO mice was significantly damaged, while the neurological function score of PLP-3 group mice was improved, indicating that neurological function was restored (Fig. 1D). After MCAO modeling, mice food and water intake were affected, resulting in weight loss and poor prognosis. During interventions on each group of mice after MCAO, the mice were weighed at the same time. MCAO mice had a significant weight loss during experiment, while PLP-3 intervention alleviated the weight loss in mice from day4 (Fig. 1E-F), indicating that the overall prognosis of mice was good.

**Fig. 2:**
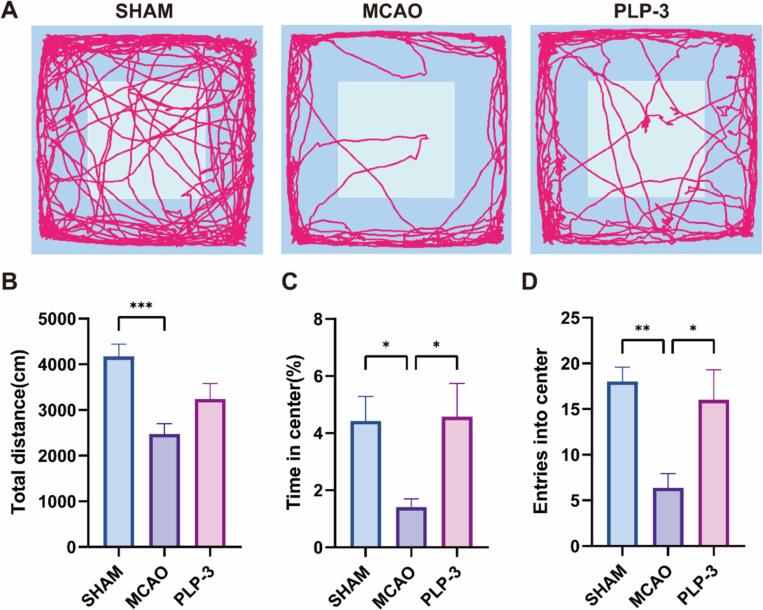
PLP-3 improves MCAO mice Neurological function (n = 8). (A) Represent image of open field test, (B) Total distance, (C)Time in center, (D) entries into center.

The open field test was conducted 7 days post-MCAO induction in mice to evaluate their anxiety-like behavior following MCAO (Fig. 2A). Results demonstrated a significant reduction in the total distance traveled by MCAO mice, as well as decreased entries into and time spent within the central zone of the open field. In contrast, after PLP-3 intervention, the time mice spent in the central zone and the number of entries into the central zone were significantly increased compared with MCAO mice. The total distance traveled by the mice tended to recover, but the difference was not statistically significant (Fig. 2B-D). These findings suggested that PLP-3 may mitigate anxiety-like behavior and improve neurological function in mice after MCAO.

*Pueraria lobata* extraction, puerarin, and resistant starch have been reported to have favorable therapeutic outcomes in animal models of cerebral ischemic injury[29], [36]. This study further demonstrates that *Pueraria lobata* polysaccharides PLP-3 also exerts an ameliorative effect on cerebral ischemic injury. Xie et al. extracted polysaccharides from *Acanthopanax* with a molecular weight of 70 kDa, which were mainly composed of glucose and galactose, the glucose content of these polysaccharides was 35.9 %. After gavage intervention, *Acanthopanax* polysaccharides significantly improved cerebral infarction and cerebral edema in MCAO rats[37]. Our study demonstrated that PLP-3 also exerts a significant improvement effect on cerebral ischemic mice, which may be attributed to its higher glucose content.

### 3.2 PLP-3 regulates neuronal and systemic inflammation in stroke mice

**Fig. 3:**
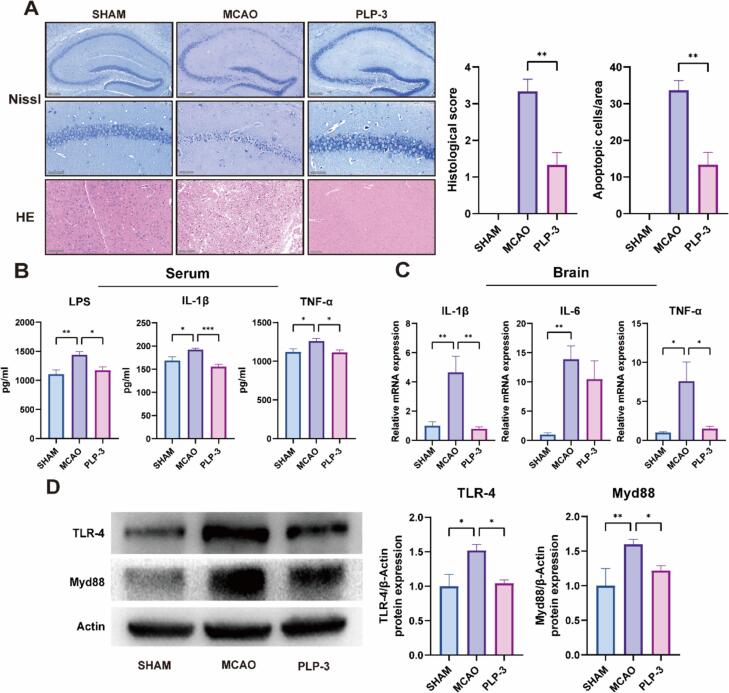
PLP-3 regulates neuronal and systemic inflammation in stroke mice. (A) Nissl and HE staining of brains in each group (n = 3, scale bar: 50 μm ∼ 200 μm), (B) serum inflammatory factors level (n = 8), (C) brain inflammatory factors mRNA expression (n = 5), (D) TLR4-Myd88 protein expression (n = 3).

When experienced cerebral ischemia and hypoxia, MCAO mice exhibited significant post-ischemic neuronal apoptosis and microglia-mediated neuroinflammation in the brain, the Nissl bodies in the cell are significantly reduced, and the nucleus of the neuron collapses[38]. The effects of PLP-3 on neuronal apoptosis and inflammation in the brains of MCAO mice were evaluated histologically using Nissl and HE staining methods. Nissl staining showed a significant reduction in Nissl bodies and significant neuronal damage in the hippocampus of MCAO mice. HE staining revealed a loss of normal tissue structure in the infarcted area and extensive infiltration of inflammatory cells. After PLP-3 intervention, the number of Apoptosis neurons in brain was significantly decreased, the pathological changes in the brain were alleviated, and the blood–brain barrier was repaired (Fig. 3A).

LPS, known as endotoxin, is a complex lipid molecule located within the cell walls of Gram-negative bacteria. LPS exerts its effects primarily through binding to TLR4, which triggers the downstream signaling pathway. This activation leads to the transcription and translation of inflammatory factors, such as IL-1β and TNF-α, ultimately promoting the inflammatory response[39]. ELISA assays revealed significantly elevated levels of LPS, TNF-α, and IL-1β in the serum of MCAO mice compared with the sham group. Serum LPS levels increased to 1439.5 pg/mL after MCAO modeling, indicating increased gut leakage. Notably, PLP-3 intervention significantly reduced LPS content in MCAO mice by 19 %. Additionally, TNF-α and IL-1β levels were also reduced by 11.9 % and 19.1 %, respectively, ameliorating systemic inflammation in MCAO mice (Fig. 3B).

Next, we detected the expression levels of TLR4 and Myd88 in the brains of mice in each group by Western blotting, the results demonstrated that the expressions of TLR4 and Myd88 in the brains of MCAO mice were significantly upregulated, exhibiting 1.58- and 1.59-fold increases compared to the SHAM group, respectively. These results suggest that the elevated LPS concentration activated the TLR4 signaling pathway in the brain. However, PLP-3 intervention significantly reduced the expression of proteins in this pathway. Following, the expression levels of inflammatory factors IL-1β, IL-6, and TNF-α by qPCR. The expressions of IL-1β and TNF-α genes in the brains of MCAO mice were significantly upregulated, while PLP-3 intervention reduced the expression of these genes. IL-6 showed a downward trend, but no significant difference was observed (Fig. 3C-D). In summary, after PLP-3 intervention, the TLR4 pathway in the brains of MCAO mice was inhibited by reducing the LPS content in the blood, thereby alleviating the inflammatory level of serum and brain tissue.

### 3.3 PLP-3 protects against intestinal barrier damage in stroke mice

Disruption of the intestinal barrier following stroke not only compromises the integrity of the intestine itself but also exerts systemic effects, influencing the homeostasis and normal function of distant organs such as immune system[40], and nervous system[41]. Plant-extracted polysaccharides have been reported to maintain mechanical barriers by enhancing intestinal mucosal barriers and upregulating the expression of tight junction proteins[21]. Therefore, we investigated the protective effects of PLP-3 on mucosal and mechanical barriers.

**Fig. 4:**
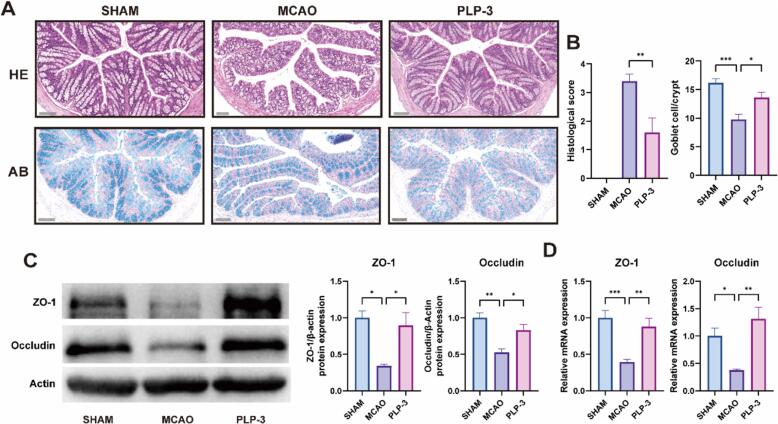
PLP-3 protects against intestinal barrier damage in MCAO mice. (A) (B)Alcian blue and HE staining of colons in each group (n = 3, scale bar: 100 μm), (C) protein expression of ZO-1 and Occludin (n = 3), (D) mRNA expression of ZO-1 and Occludin (n = 5).

Histopathological examination using HE staining revealed that the intestinal epithelial tissue of MCAO group mice exhibited structural damage, including loss of normal structure, necrosis and shedding of intestinal epithelial cells, decreased crypt depth, and inflammatory cell infiltration. However, PLP-3 intervention ameliorated these pathological changes, and the intestinal tissue exhibited a tendency towards normal morphology. Alcian blue staining specifically stains acidic mucin in intestinal goblet cells[42]. Quantification of goblet cells in the ileum and colon revealed a significant reduction in MCAO mice, indicating damage to the intestinal mucosal barrier after cerebral ischemia. PLP-3 intervention significantly increased the number of goblet cells and repaired the damaged intestinal mucosa (Fig. 4A-B).

Intestinal tight junction proteins, such as the ZO family and Occludin, are junction proteins located between intestinal epithelial cells. Their expression levels reflect the size of the intestinal gap and play a crucial role in maintaining the mechanical barrier of the intestine[43]. Western blot and qPCR experiments were conducted to assess the protein and mRNA expression levels of ZO-1 and Occludin. The results demonstrated that MCAO mice exhibited not only pathological alterations but also decreased expression of ZO-1 and Occludin. Compared to the SHAM group, the expression levels of ZO-1 and Occludin were downregulated by 66 % and 48 %, respectively (Fig. 4C-D). This suggests the occurrence of intestinal leakage, which facilitates the entry of harmful bacterial substances, such as LPS, into the bloodstream (as previously validated). Conversely, PLP-3 intervention upregulated the expression of ZO-1 and Occludin, thereby rescuing the integrity of the intestinal barrier. At the mRNA level, the qPCR results corroborated these findings, demonstrating a similar significant trend. Notably, PLP-3 intervention not only repaired the intestinal mucosal barrier but also maintained the mechanical barrier of the intestine in a stable state.

### 3.4 PLP-3 modulates the composition and structure of the gut microbiota

**Fig. 5:**
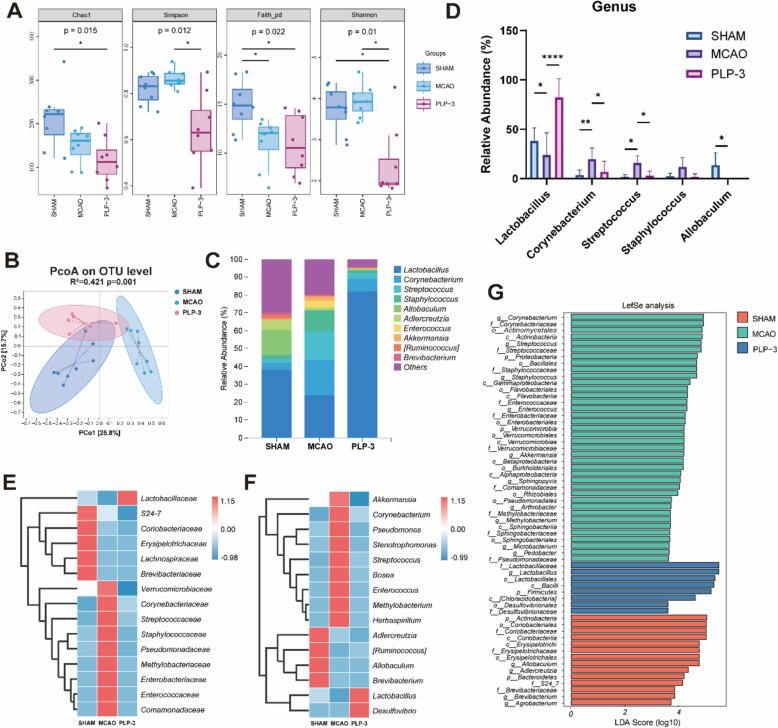
Effect of PLP-3 on composition and structure of the gut microbiota (n = 8). (A) α-diversity, (B) PcoA analysis, (C) Species composition analysis at the genus levels, (D) Comparison of the abundance of the top five enriched genus, (E)(F) Species composition heatmap at family and genus level, (G) Lefse analysis.

Ischemic stroke has been associated with rapid dysbiosis of the gut microbiota, primarily characterized by the enrichment of pathogen such as LPS producer *Enterobacteriaceae,* opportunistic pathogens *Erysipelotrichales* and *Staphylococcus.* [15], [40]. Prebiotics interventions targeting the gut microbiota have demonstrated significant neuroprotective effects in models of cerebral ischemia[44], [45]. The effects of PLP-3 on the gut microbiota of MCAO mice were evaluated using 16S rRNA sequencing. The Faith-PD index of the MCAO group was significantly lower than that of the SHAM group (Fig. 5A), indicating reduced gut microbiota diversity in MCAO mice. Notably, the PLP-3 group exhibited significantly lower diversity indices compared to both the SHAM and MCAO groups. This suggests that PLP-3 homogenizes the gut microbiota, leading to decreased diversity (Fig. 5A). Principal co-ordinates analysis (PCoA) based on bray-curtis distances revealed distinct clustering of the gut microbiota among the three groups (Fig. 5B). These findings indicate substantial alterations in the gut microbiota of MCAO mice, characterized by a marked decline in both diversity and species abundance. Additionally, PLP-3 treatment modified the gut microbiota structure and composition of MCAO mice.

Species composition analysis showed an enrichment of *Corynebacterium*, *Streptococcus*, and *Staphylococcus* at the genus level (Fig. 5C). These bacteria are frequently associated with opportunistic infections and have been linked to host diseases in the context of conditions such as low immunity and intestinal barrier damage[46]. Members of the *Corynebacterium* genus are increasingly recognized as opportunistic pathogens, causing disease in specific circumstances, such as in immunocompromised individuals or patients with prosthetic devices[46]. *Staphylococcus* is a common pathogen genus that primarily forms biofilm and produces enterotoxins in vivo, leading to the development of purulent inflammatory diseases[47]. Additionally, studies have demonstrated that *Staphylococcus aureus* can impair insulin function via outer membrane proteins, contributing to the acceleration of obesity and hyperlipemia[48]. PLP-3 intervention led to a reduction in the abundance of opportunistic pathogens *Corynebacterium* and *Staphylococcus*. Interestingly, PLP-3 enriched *Lactobacillaceae* and *Lactobacillus* at both family and genus level, accounting for 82.15 ± 19.09 % of the microbial composition, and this difference was statistically significant (Fig. 5D-F). The high prevalence of *Lactobacillus* may explain the decrease in gut microbiota species diversity after PLP-3 intervention, as the enrichment of beneficial bacteria likely suppressed the growth of opportunistic pathogens. Differential bacteria between groups were identified using Linear Discriminant analysis Effect Size assay (Lefse), corroborating that the PLP-3 group restored the microbial dysbiosis in MCAO mice by promoting *Lactobacillus* and suppressing opportunistic pathogens (Fig. 5G).

Previous studies have shown that oral administration of *Lactobacillus* has a significant improvement effect on mice with cerebral ischemia models[24]. However, the colonization rate is affected by digestive fluids and individual differences. Polysaccharides have good digestive resistance and is only utilized by microorganisms in the intestine. PLP-3 can enrich *Lactobacillus* in vivo, thereby improving the intestinal microecology of opportunistic pathogens after stroke. Therefore, PLP-3 has the potential to become an effective targeted intervention for gut microbiota, offering new treatment options in the future.

### 3.5 PLP-3 enriches SCFAs

SCFAs are metabolites produced by gut microbiota through the degradation and utilization of dietary fibers and serve as direct energy source for intestinal epithelial cells, sodium butyrate supplementation alleviates intestinal barrier damage, after entering the bloodstream, it improves neuroinflammation in MCAO rats by binding to GPR41 and regulating the PI3K/AKT pathway[49]. Furthermore, butyrate treatment of db/db mice fecal transplantation can mitigate ischemic stroke injury[15]. Additionally, acetate and propionate can regulate lipid metabolism and antioxidative stress[50].

**Fig. 6:**
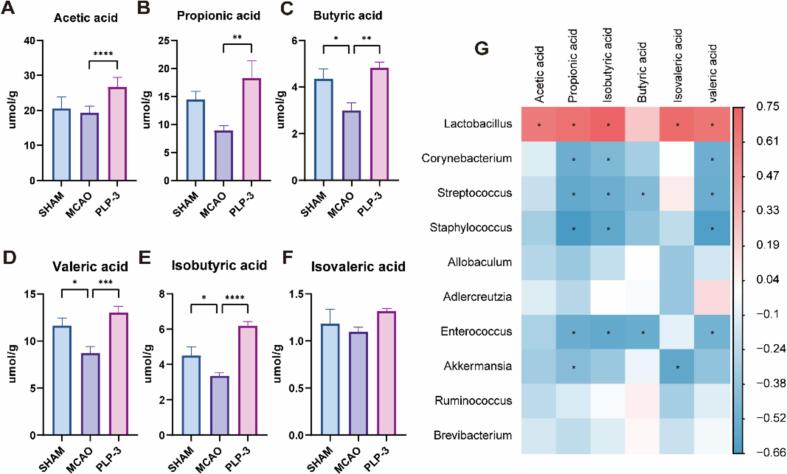
Effect of PLP-3 on feces SCFAs profile (n = 8). (A) Acetic acid, (B) Propionic acid, (C) Butyric acid, (D) valeric acid, (E) Isobutyric acid, (F) Isovaleric acid, (G) Spearman correlation analysis on SCFAs and the top ten enriched genus.

Using gas chromatography-mass spectrometry (GC–MS), we conducted a targeted SCFAs profile analysis on feces of mice in each experimental group. The analysis included acetic acid, propionic acid, butyric acid, isobutyric acid, valeric acid, and isovaleric acid. The results (Fig. 6) showed that, compared with the SHAM group mice, the levels of butyric acid, isobutyric acid, and valeric acid in the MCAO group mice were significantly decreased. Conversely, the levels of acetic acid, propionic acid, and isovaleric acid showed a downward trend, but the differences were not significant, indicating that MCAO significantly disrupts SCFAs metabolism in mice, which is consistent with previous reports[51]. Especially, the content of acetic acid, propionic acid, butyric acid, isobutyric acid and valeric acid in the PLP-3 group reached 24.14 ± 0.79, 22.21 ± 2.64, 4.76 ± 0.25, 6.20 ± 0.23 and 13.05 ± 0.66 µmol/g, which were 1.18, 1.53, 1.08, 1.38 and 1.12 times the SHAM group, and 1.30, 2.48, 1.57, 1.84 and 1.56 times the MCAO group, respectively. Next, we performed spearman correlation analysis on SCFAs and the top ten enriched bacteria genus (Fig. 6G). The results showed that, except for butyric acid, the *Lactobacillus* genus was positively correlated with the levels of all elevated SCFAs, and the differences were statistically significant. Conversely, *Corynebacterium*, *Streptococcus*, and *Staphylococcus* were significantly negatively correlated with the levels of some SCFAs.

Studies have shown that in vivo intervention of dietary polysaccharides can significantly increase the levels of SCFAs in mice, which is an important mechanism by which polysaccharides exert their prebiotic effects[35]. Our findings demonstrate that PLP-3 can significantly increase the levels of acetic acid, propionic acid, butyric acid, and valeric acid in MCAO mice, indicating its high bioavailability. However, in vitro experiments are still needed to provide auxiliary evidence for this claim.

### 3.6 PLP-3 modulate the metabolism profile of MCAO mice

The metabolites of gut microbiota are key pathways through which gut microbiota contributes to the progression of stroke disease. In patients with acute ischemic stroke, Zhang et al. observed significant bile acid disorders, characterized by a marked reduction in ursodeoxycholic acids[32]. Additionally, the accelerated degradation of tryptophan to kynurenine after stroke was positively correlated with post-stroke inflammation[52]. To elucidate the effects of PLP-3 on gut microbiota-related metabolites in mice after MCAO, we performed a non-targeted metabolomics study of mouse cecum content using LC-MS. Orthogonal partial least squares-discriminant analysis (OPLS-DA) revealed significant differences in the metabolomic profiles of MCAO and PLP-3 groups. As visualized in volcano plots, based on filtering criteria of variable importance in projection (VIP) > 1, and p < 0.05, PLP-3 upregulated 78 metabolites and downregulated 58 metabolites compared to the MCAO group.

**Fig. 7:**
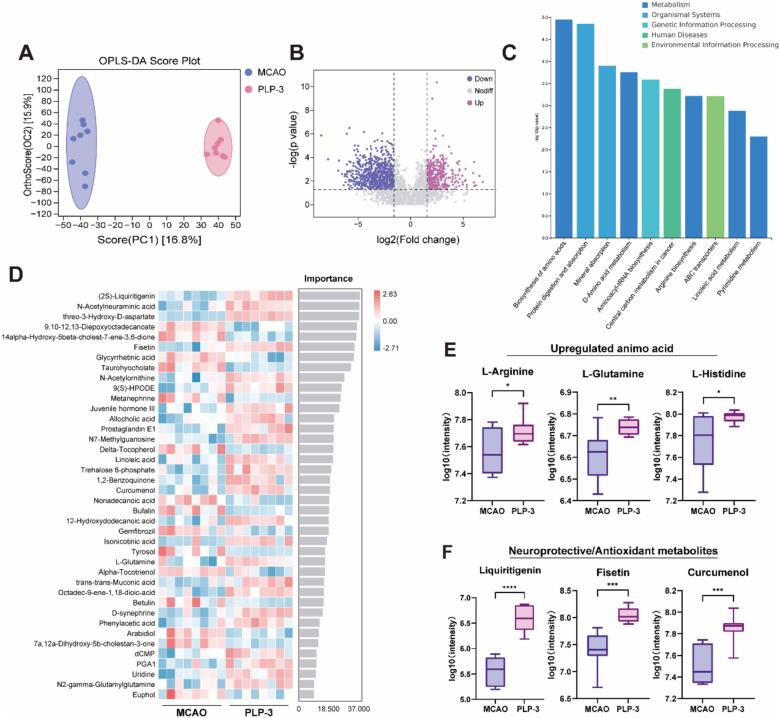
PLP-3 modulate the cecal metabolism profile of MCAO mice (n = 8). (A) OPLS-DA analysis, (B) Volcanic diagram of differential metabolites, (C) KEGG pathway upregulated by PLP3, (D) Random forest analysis of differential metabolites, (E) represent upregulated animo acid, (F)upregulated neuroprotective/antioxidant metabolites.

All differential metabolites were included in subsequent Kyoto Encyclopedia of Genes and Genomes (KEGG) enrichment analysis, which identified differences in metabolic profiles between MCAO and PLP-3 groups primarily in the following pathways: Biosynthesis of amino acids, Protein digestion and absorption, mineral absorption, D-amino acid metabolism, Aminoacyl-tRNA biosynthesis, Central carbon metabolism in cancer, Arginine biosynthesis et al (Fig. 7C). Through random forest analysis, the importance of differential metabolites for the classification model was scored. A heat map was generated to visualize the abundance distribution of these metabolites in each sample, metabolites with high importance can be considered marker metabolites for intergroup differences (Fig. 7D).

Researches have shown that supplementing amino acids and regulating amino acid metabolism can repair damaged intestinal barriers and regulate intestinal immune function[53], [54], [55]. The enrichment of the “Biosynthesis of amino acids” and “Amino acid metabolism” pathway in KEGG indicates that PLP-3 intervention regulates amino acid metabolism and enhanced amino acid biosynthesis in the intestines of MCAO mice. Further analysis of the significantly elevated amino acids in amino acid metabolism revealed that PLP-3 increased the levels of glutamine, arginine, and histidine in the cecum (Fig. 7E), which may alleviate intestinal inflammation after stroke, promote the differentiation of intestinal goblet cells, and protect the intestinal mucosa of mice after stroke.

**Fig. 8:**
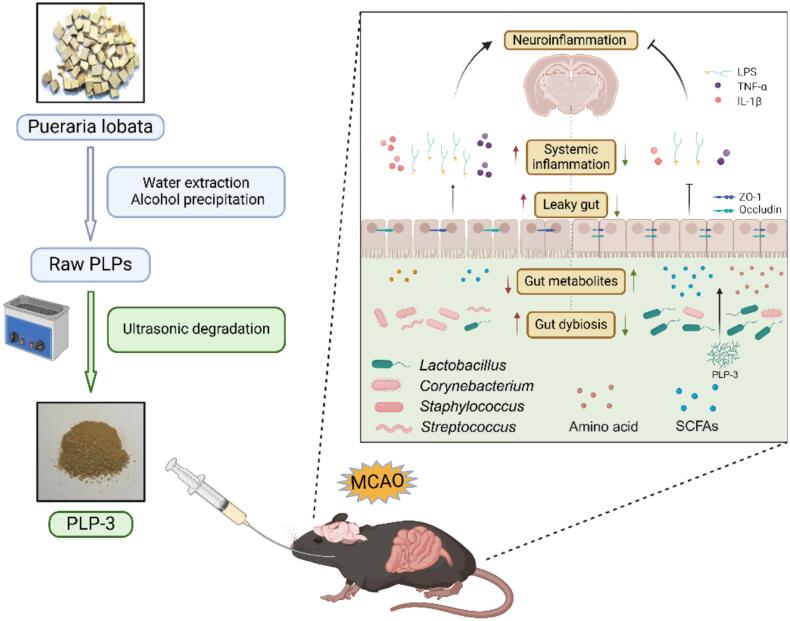
Schematic of mechanism in PLP-3 against ischemic brain injury by modulating gut microbiota and LPS-TLR-4 pathway.

Additionally, we found metabolites with neuroprotective and antioxidant activities in the upregulated metabolites (Fig. 7F), such as the flavonoids Liquiritigenin and fisetin, which have been reported to have good application value in anti-oxidative stress and cardiovascular diseases[56], [57]. Curcumenol is also a plant-derived pharmaceutical ingredient that can inhibit the NF-κB pathway[58]. This suggests that PLP-3 supplementation after MCAO not only improves the gut microbiota, but also alters the intestinal metabolic profile of mice, regulates amino acid metabolism, and enriches neuroprotective beneficial metabolites, further supporting the evidence of PLP-3 as a dietary prebiotic, the mechanism of PLP-3 targeting the gut-brain axis is illustrated in Fig. 8.

## 4 Conclusion

In this study, we demonstrated that *Pueraria lobata* polysaccharide PLP-3, prepared by ultrasound-assisted extraction, could enrich probiotics such as *Lactobacillus* in the intestine, thereby increasing the concentration of SCFAs and improving the intestinal mucosal and mechanical barriers. By reducing the entry of LPS into the blood and inhibiting the activation of TLR-4 in the brain, PLP-3 can alleviate inflammatory levels in brain tissue and the whole body, ultimately improving the recovery of cerebral infarction and neurological function in MCAO mice. In conclusion, ultrasound-degraded PLP-3 is prebiotic, which can regulate gut microbiota and metabolites to play a multi-pathway and multi-target role in cerebral ischemia improvement, providing a theoretical basis for their use as an intervention strategy targeting the brain-gut axis.

## CRediT authorship contribution statement

**Yulong Zhang:** Writing – original draft. **Zuman Dou:** Formal analysis, Conceptualization. **Shanshan Li:** Software, Resources, Conceptualization. **Huaying Zhang:** Data curation. **Shanshui Zeng:** Formal analysis, Data curation. **Xiangyu Zuo:** Formal analysis. **Yu Xiao:** Data curation. **Lingling Zhang:** Investigation, Formal analysis. **Zhixin Li:** Methodology. **Qingfeng Zhu:** Data curation. **Wenyang Zhang:** Formal analysis, Data curation. **Hui Niu:** Methodology, Investigation, Formal analysis, Conceptualization. **Qingfei Duan:** Methodology, Investigation. **Xiaoxia Chen:** Investigation, Methodology. **Zhuang Li:** Software, Resources, Methodology, Investigation. **Hongwei Zhou:** Visualization, Supervision, Resources, Conceptualization. **Qian Wang:** Writing – review & editing, Supervision, Resources, Funding acquisition.

## Declaration of competing interest

The authors declare that they have no known competing financial interests or personal relationships that could have appeared to influence the work reported in this paper.
